# Traumatic Injuries in Sexual Assault Patients in the Emergency Department

**DOI:** 10.5811/westjem.2022.1.53994

**Published:** 2022-08-19

**Authors:** Denise McCormack, Sushi Subburamu, Glenda Guzman, Carmen Calderon, Ruchika Darapaneni, Robert Lis, Niloofar Sima, Jeremy Sperling, Jill Corbo

**Affiliations:** * Albert Einstein College of Medicine, Department of Emergency Medicine, Bronx, New York; † Jacobi Medical Center and North Central Bronx Hospital, Department of Emergency Medicine, Bronx, New York; ‡ Montefiore and Jacobi Medical Center, Department of Emergency Medicine, Bronx, New York; § North Central Bronx Hospital, Department of Social Work, Bronx, New York; ¶ Albert Einstein College of Medicine, Office of Medical Student Research, Bronx, New York

## Abstract

**Introduction:**

The emergency department (ED) is at the forefront for treatment of sexual assault patients. Many require treatment for injuries sustained during the assault, ranging from mild to severe. Our objective in this study was to characterize types of injuries associated with sexual assault and identify associated factors.

**Methods:**

We reviewed ED charts from an inner-city trauma center and nearby community hospital from 2019–2020 for patients age ≥13 years with a chief complaint of sexual assault. We used descriptive statistics, chi square, and logistic regression to characterize demographics and identify factors associated with trauma.

**Results:**

A total of 157 patients met inclusion criteria. The mean age was 27.9 years old (range 13–79 years) and 92.4% were female. Adult patients (age >18 years) comprised 77.5% of assaults vs adolescents (age 13–18 years) at 22.3%. Most patients presented to the trauma center compared to the community hospital (69.4% vs 30.6%). The assailants were reported as 61.2% acquaintance, 22.9% stranger, and 15.9% intimate partner. A forensic rape kit was performed in 92 (58.6%) cases. The patient was intoxicated with alcohol in 39 (24.8%) cases, and 22 (14%) patients reported drug-facilitated assault where an unknown substance was given to them. Alcohol (P = 0.95) and drug-facilitated assault (P = 0.64) did not change the occurrence of injuries. Fifty-seven (36.3%) patients exhibited physical trauma on presentation. Forty-five (28.6%) patients had minor injuries of abrasions, lacerations, or contusions. Major trauma was defined as fracture, brain injury, hemorrhage, strangulation, or injury requiring surgical consultation. There were 12 patients with major trauma consisting of fracture injury or nonfatal strangulation. None of the patients required admission. Sexual assault by an intimate partner (odds ratio [OR] 2.6; 95% CI: 1.1–6.5) and being an adult patient compared to adolescent (OR 3.0; 95% CI, 1.1–7.7) was significantly associated with physical trauma. Sexual assault by an intimate partner was also associated with nonfatal strangulation (OR 4.0; 95% CI, 1.1–15.4).

**Conclusion:**

Physical injuries that resulted from sexual assault were mostly minor and occurred in 36% of rape victims. Intimate partner violence was found to be associated with physical trauma as well as nonfatal strangulation. Overall, this study helps us to understand key factors associated with sexual violence.

## INTRODUCTION

In the United States, approximately 52 million women and 27 million men have experienced sexual assault (SA) in their lifetime.^1^ The emergency department (ED) remains the most common place where SA patients first seek out comprehensive care to receive emergency contraception, prophylaxis against sexually transmitted infections, completion of a forensic rape kit, and treatment for their injuries. Studies have shown that 30–80% of SA patients present to the ED with traumatic injury.^2^–^4^ However, there is conflicting evidence regarding the severity of these injuries.^5^–^6^

Several prior studies suggested that traumatic injuries during sexual assault were more likely to occur when a stranger was the assailant.^7^–^9^ However, other studies determined that a significant injury was more likely to happen when the assailant was an intimate partner (IP).^10^–^11^ In this study we evaluated the likelihood of SA being committed by an IP, acquaintance, or stranger, and whether this was related to the patient experiencing traumatic injuries.

Sexual assaults are frequently associated with drug-facilitated sexual assault (DFSA), illicit drugs, or alcohol. Drug-facilitated sexual assault has prevalence as high as 20.9% and is defined as when a drug is given to incapacitate the victim. Common DFSA drugs are gamma hydroxybutyrate, ketamine and benzodiazepines.^12^–^15^ Over-the-counter agents such as diphenhydramine (Benadryl) and Visine eye drops have also been reported.^16^–^17^ Alcohol intoxication in comparison to DFSA is more frequent and is typically the most common substance associated with sexual assault, occurring in 33–60% of cases.^18^–^19^ In this study, we aimed to determine how frequently SA patients sustained traumatic injuries when either alcohol or DFSA was involved.

## METHODS

### Patient Selection

We conducted a retrospective ED chart review from July 1, 2019–July 31, 2020 from a Level I trauma center with over 100,000 annual visits and community hospital with 50,000 annual visits, both located in medically underserved areas. Both hospitals are state designated Sexual Assault Forensic Examiner (SAFE) facilities of excellence with a dedicated sexual assault response team. Professionals from the team respond to all ED cases presenting with a chief complaint of SA. The team has formal training and expertise in providing standardized care to SA patients based on federal and state guidelines.^20^–^22^ The institutional review board approved this study.

### Inclusion and Exclusion Criteria

Inclusion criteria consisted of patients **≥**13 years of age with an ED chief complaint of SA. Patients were excluded if they were younger than 13, left after nursing triage assessment, or had an acute psychiatric condition based on medical history and impairment of mental status. We omitted from the study charts with missing variables of interest.

### Demographics

Adolescent was defined as age 13–18 years old and adult >18 years. Racial categories were Black, Hispanic, White, Asian and other. Adult age was divided into 19–34, 35–64, and **≥**65.

**Population Health Research Capsule**

What do we already know about this issue?

*An estimated 52 million women and 27 million men in the USA have experienced sexual assault in their lifetime. The emergency department (ED) is at the forefront for the specialized treatment of these patients*.

What was the research question?

*What are the key elements associated with ED presentations of sexual assault and traumatic injury?*

What was the major finding of the study?

*Physical trauma was found in 36.3% of sexual assault patients, with 8% categorized as major trauma. Intimate partner violence was found in 15.9% of ED complaints for sexual assault*.

How does this improve population health?

*This study helps us to understand the complexities of sexual violence with the goal of improving the patient care model for this vulnerable patient population*.

### Data Collection

Three research fellowship medical students RL, RD and NS served as abstractors and conducted supervised chart reviews, according to best practices in medical record review.^23^ The recommended chart review methods were adhered to for this study. The data abstractors received training in electronic health record (EHR) data collection. A research protocol with specific variables, standardized definitions, and abstraction procedures was provided to the three abstractors who were blinded to the hypothesis being tested. The data collection form was piloted for reliability prior to finalization. The three abstractors met with the first author on a regular basis who reviewed charts for interobserver reliability and uniformity of data collection procedures.

Eligible patients were identified by a list generated from the hospital EHR based upon a chief complaint of SA. The patient list was then confirmed with the names listed on the hospital SA hotline call log to confirm a complete consecutive patient sample. Each patient chart was reviewed for inclusion and exclusion criteria and relevant clinical details about their ED visit. The first author adjudicated all questions related to inclusion/exclusion criteria and clinical information.

### Definitions

#### Sexual Assault

The penetration, no matter how slight, of the vagina or anus with any body part or object, or oral penetration by a sex organ of another person, without the consent of the victim.

#### Emergency Medical Services (EMS) arrival

Patient arrived to the ED by ambulance or brought by local police.

#### Acquaintance

Friend, classmate, relative, neighbor, or co-worker.

#### Intimate Partner (IP)

Current or former spouse, girlfriend, boyfriend, or partner.

#### Stranger

Perpetrator who was unknown to the patient.

#### Non-fatal strangulation (NFS)

The impairment of air or blood flow through the neck as a result of external pressure. Manual or ligature strangulation performed by applying direct pressure usually with the hands around the neck or by tightening a ropelike ligature around the neck.^24^

#### Drug-facilitated sexual assault (DFSA)

Suspected if the patient remembers consuming a beverage but cannot recall what happened for a period of time after consumption or feels a lot more intoxicated than their response to the amount of alcohol consumed or feels intoxicated after drinking a non-alcoholic beverage. If the patient woke up experiencing memory lapses or was unable to account for a period of time or the patient feels as though someone had sexual intercourse with them but cannot recall any or all of the incident.^25^

#### Alcohol and illicit drug use

Patient reports consuming alcohol or using an illicit substance during the immediate time period leading up to the SA.

#### Traumatic injury

Minor injury was defined as laceration, abrasion, or contusion to general areas of the body, excluding genital trauma. Major injury was defined as fracture, traumatic brain injury, internal hemorrhage, any evidence of attempted strangulation, or any injury requiring consultation by a surgical subspecialty.

### Statistical Analysis

We used chi-square tests for statistical analysis of categorical variables: age, gender, race, involvement of alcohol, illicit drug or DFSA, perpetrator type, completion of forensic rape kit, and presence of injury on exam. Logistic regression was performed to identify associations with traumatic injury, as measured by calculated odds ratio (OR) and 95% confidence interval (CI). Statistical significance was defined as *P* < 0.05.The software program Stata version 16 (StataCorp LLC, College Station, TX) was used to compute statistical analyses.

## RESULTS

A total of 157 patients met inclusion criteria, and 15 patients were excluded from the study. Nine patients were excluded due to age <13 years, two patients were excluded because of an acute psychiatric condition, and four patients left the ED after triage assessment. The mean age was 27.9 years old (range 13–79 years), and 92.4% were female (Table 1). Adult patients (age >18 years) comprised 77.5% of assault victims compared to adolescents (age 13–18 years) at 22.3%. Most patients presented to the trauma center compared to the community hospital (69.4% vs 30.6%). The perpetrators of these assaults were reported as 61.2% acquaintance, 22.9% stranger, and 15.9% IP. In 8.9% of cases, there was an assault by multiple assailants. Fifty-seven (36.3%) patients exhibited traumatic injury on presentation. A forensic rape kit was performed in 92 (58.6%) cases but was not associated with the presence of trauma (*P* = 0.23) (Table 2).

There were 39 (24.8%) cases where the patient was intoxicated with alcohol (*P* = 0.95), and 22 (14%) reported DFSA (*P* = 0.64), but neither was associated with physical trauma (Table 2). Forty-five (28.6%) patients had minor injury described as abrasions, lacerations or contusions, and major trauma occurred in 12 patients, which consisted of either having a fracture injury or NFS. Logistic regression determined that sexual assault by IP (OR 2.6; 95% CI: 1.1–6.5) and being an adult patient compared to adolescent (OR 3.0; 95% CI: 1.1–7.7) was associated with physical injury (Table 3). A sexual assault perpetrated by IP also was associated with NFS (OR 4.0; 95% CI: 1.1–15.4).

## DISCUSSION

Our results show that SA patients who present to the ED for treatment are overwhelmingly young women and their acquaintances are commonly the perpetrator. This familiar pattern may help to explain why under-reporting to law authorities might occur.^26^–^27^ The United States Department of Justice estimates that up to 67% of sexual assaults are not reported to the police.^28^ In our study we discovered that merely 58.6% of ED patients consented to have a forensic rape kit completed, a process that entails collecting DNA evidence from the patient to aid in the legal prosecution of a perpetrator. In SA cases where the patient declined a forensic rape kit in the ED, the assault was not disclosed to law enforcement officials at the patient’s request. Although our study did not examine the reasons why patients declined a forensic rape kit, it has been widely reported that many patients do not report SA to law enforcement due to fear of reprisal, shame, and stigma.^29^–^30^

The physical injuries of the SA patients were typically mild, and none of the patients were admitted. Patients with physical trauma were more often adults than adolescents. The reason for this finding is unknown. We found alcohol to be the most common substance associated with SA, whereas DFSA and illicit drug use were relatively low. Furthermore, neither alcohol nor DFSA were associated with trauma. This differs from prior research where patients with DFSA were less likely to sustain physical injuries during SA, possibly due to sedation and lessened mobility.^31^–^32^

When major trauma resulted, specifically NFS or “choking,” the perpetrator was often an IP, consistent with previous studies.^33^–^36^ Our finding that SA patients were more likely to experience attempted strangulation if the perpetrator was an IP is alarming, since this carries an increased homicide risk.^34^ However, the occurrence of NFS was relatively low at 6.4% in our patient sample, which is similar to a large study that found NFS in 7.4% of SA cases.^33^ It remains difficult to determine whether this occurrence is low because some strangulation victims did not survive.

Our findings confirm the conclusions of previous research that severe trauma in SA victims is infrequent. However, we discovered that there was more trauma associated with IP sexual violence (IPSV), which occurred in 15.9% of our inner-city patients. It is an often overlooked problem even though IPSV occurs in 1/10 men and 1/4 women nationwide.^1^,^37^ A recent multicenter study found that 11.4% of patients who presented to Level I trauma centers for injury also reported IPSV.^38^ Additional research is needed to determine whether the prevalence of IPSV is greater among inner-city ED patients compared to other regions. This discovery could lead to improved SA protocols and resource allocation in higher risk communities.

Overall, it remains imperative that clinicians in the ED adhere to screening guidelines for intimate partner abuse and address the topics of SA and IPSV in the ED when applicable. Tangible solutions to these challenges are still evolving; nevertheless, our study results can be used to enhance education of medical professionals with an emphasis on improving standardized care for SA victims and optimizing forensic rape kit collection when required.

## LIMITATIONS

Our study was retrospective and drew patients from EDs designated as SAFE facilities of excellence located in an inner-city community. Therefore, the results may not be generalizable to other communities. Additionally, the patient sample was generated from the EHR based on a chief complaint of SA, which may have underestimated the true occurrence of SA in our patients due to selection and sample biases. The study sample could also have missed patients with a primary trauma-related complaint or those with altered mental status, where the SA was either addressed secondarily or never disclosed to the treatment team. Our study most likely did not capture cases in which a patient presented in extremis due to severe trauma and the SA aspect was unknown to the ED clinicians. In addition to these several factors, we recognize that SA is often under-reported, which further contributed to our study having a small number of patients.

## CONCLUSION

Alcohol is the most common substance that is reported among sexual assault patients presenting to the ED. Traumatic injuries occurred in just more than one-third of these SA victims and were categorized as minor. Intimate partner sexual violence was found to be substantially associated with physical trauma and strangulation. Overall, this study helps us to understand several key factors associated with sexual violence.

## Figures and Tables

**Table 1:** Sexual assault patient characteristics.

Patient characteristics	Total N = 157
Age	
27.9 years ± 11.5	
13–18 years adolescent	35, 22.3%
≥19 years adult	122, 77.7%
Adult	
19–34 years	82, 67.2%
35–64 years	38, 31.1%
≥ 65 years	2, 1.7%
Gender	
Female	145, 92.4%
Male	12, 7.6%
Race	
Hispanic	75, 47.7%
Black	51, 32.5%
White	22, 14%
Asian	7, 4.5%
Other	2, 1.3%
Perpetrator	
Acquaintance	96, 61.2%
Stranger	36, 22.9%
Intimate partner	25, 15.9%
DFSA	
Yes	22, 14.0%
No	135, 86.0%
Alcohol-related	
Yes	39, 24.8%
No	118, 75.2%
Illicit drug	
Yes	6, 3.8%
No	151, 96.2%
Mode of arrival	
EMS	89, 56.7%
Walk-in	68, 43.3%
SAFE Facility	
Level I trauma center	109, 69.4%
Community hospital	48, 30.6%
Forensic rape kit	
Yes	92, 58.6%
No	65, 41.4%
Traumatic Injury	
Yes	57, 36.3%
Major	12, 21.1%
Minor	45, 78.9%
No	100, 63.7%
Non-fatal strangulation	
Yes	10, 6.4%
No	147, 93.6%

*DFSA*, drug-facilitated sexual assault; *EMS*, emergency medical services

*SAFE*, Sexual Assault Forensic Examiner.

**Table 2:** Comparison of sexual assault patients who suffered trauma.

Patient characteristics	All N = 157	Trauma n = 57	No trauma n = 100	X^2^ P-value
Age				
Adolescent	35	6, 17.1%	29, 82.9%	*P<0.05
Adult	122	51, 41.8%	71, 58.2%	
Adult				
19–34 years	82	31, 37.8%	51, 62.2%	0.15
35–64 years	38	18, 47.4%	20, 52.6%	
≥ 65 years	2	2, 100%	0, 0%	
Gender				
Female	145	54, 37.2%	91, 62.8%	0.69
Male	12	3, 25%	9, 75%	
Race				
Hispanic	75	29, 38.7%	46, 61.3%	0.75
Black	51	17, 33.3%	34, 66.7%	
White	22	9, 40.9%	13, 59.1%	
Asian	7	2, 28.6%	5, 71.4%	
Other	2	0, 0%	2, 100%	
Perpetrator				
Acquaintance	96	24, 25%	72, 75%	*P<0.05
Stranger	36	18, 50%	18, 50%	
Intimate partner	25	15, 60%	10, 40%	
DFSA				
Yes	22	7, 31.8%	15, 68.2%	0.64
No	135	50, 37.1%	85, 62.9%	
Alcohol-related				
Yes	39	14, 35.9%	25, 64.1%	0.95
No	118	43, 36.4%	75, 63.6%	
Illicit drug				
Yes	6	2, 33.3%	4, 66.7%	0.88
No	151	55, 36.4%	96, 63.6%	
SAFE facility				
Level I trauma center	109	38, 34.9%	71, 65.1%	0.57
Community hospital	48	19, 39.6%	29, 60.4%	
Forensic rape kit				
Yes	92	37, 40.2%	55, 59.8%	0.23
No	65	20, 30.8%	45, 69.2%	

DFSA, drug-facilitated sexual assault; *SAFE*, Sexual Assault Forensic Examiner.

**Table 3:** Associations of sexual assault with traumatic injury.

Predictor	Odds Ratio; 95% CI
Adult patient > 18 years old	3.0; CI: 1.1 – 7.7
Intimate partner violence	2.6; CI: 1.1 – 6.5
